# High levels of circulating endothelial progenitor cells in patients with diabetic retinopathy are positively associated with ARHGAP22 expression

**DOI:** 10.18632/oncotarget.24909

**Published:** 2018-04-03

**Authors:** Yu-Chuen Huang, Wen-Ling Liao, Jane-Ming Lin, Ching-Chu Chen, Shih-Ping Liu, Shih-Yin Chen, Yu-Ning Lin, Yu-Jie Lei, Huan-Ting Liu, Yu-Jen Chen, Fuu-Jen Tsai

**Affiliations:** ^1^ Department of Medical Research, China Medical University Hospital, Taichung 404, Taiwan; ^2^ Center for Personalized Medicine, China Medical University Hospital, Taichung 404, Taiwan; ^3^ Department of Ophthalmology, China Medical University Hospital, Taichung 404, Taiwan; ^4^ Division of Endocrinology and Metabolism, China Medical University Hospital, Taichung 404, Taiwan; ^5^ Center for Translational Medicine, China Medical University Hospital, Taichung 404, Taiwan; ^6^ Children's Hospital of China Medical University, Taichung 404, Taiwan; ^7^ Department of Medical Genetics, China Medical University Hospital, Taichung 404, Taiwan; ^8^ School of Chinese Medicine, China Medical University, Taichung 404, Taiwan; ^9^ Graduate Institute of Integrated Medicine, China Medical University, Taichung 404, Taiwan; ^10^ Graduate Institute of Biomedical Science, China Medical University, Taichung 404, Taiwan; ^11^ Department of Radiation Oncology, Mackay Memorial Hospital, Taipei 104, Taiwan; ^12^ Department of Medical Research, Mackay Memorial Hospital, New Taipei City 251, Taiwan; ^13^ Institute of Traditional Medicine, National Yang-Ming University, Taipei 112, Taiwan; ^14^ Department of Social Work, Asia University, Taichung 413, Taiwan; ^15^ Department of Biotechnology, Asia University, Taichung 413, Taiwan

**Keywords:** type 2 diabetes, diabetic retinopathy, circulating endothelial progenitor cells, ARHGAP22

## Abstract

Diabetic retinopathy (DR) is a common microvascular complication of diabetes. Circulating endothelial progenitor cells (EPCs) are derived from bone marrow and are characterized by pathological retinal neovascularization. *Rho GTPase Activating Protein 22* (*ARHGAP22*) is a DR susceptibility gene that interacts with its downstream regulatory protein ras-related C3 botulinum toxin substrate 1 (Rac1), to assist in endothelial cell angiogenesis and increasing capillary permeability. The aim of this study was to elucidate the relationship between ARHGAP22 expression and EPC levels in type 2 diabetes (T2D) patients with DR. Fifty T2D patients with DR were recruited. Circulating EPCs were characterized as CD31^+^/vascular endothelial growth factor-2^+^/CD45^dim^/CD133^+^ and were quantified using triple staining flow cytometry. Real-time polymerase chain reaction tests were used to quantify ARHGAP22 expression. We found that T2D patients with proliferative DR had significantly lower EPC levels than those with non-proliferative DR (*P* = 0.028). T2D patients with EPC levels above the median value (> 4 cells/10^5^ events) had higher levels of ARHGAP22 expression (*P* = 0.002). EPC levels were positively correlated with ARHGAP22 expression (*r* = 0.364, *P* = 0.009). Among T2D patients with DR, a higher expression of ARHGAP22 was associated with higher levels of EPCs. ARHGAP22 may be involved in the mobilization or active circulation of EPCs, thus contributing to neovascularization during DR development.

## INTRODUCTION

Diabetic retinopathy (DR) is a common microvascular complication of diabetes and remains a leading cause of visual loss in developing countries among working age individuals [1]. Poor glycemic control, longer duration of diabetes, hypertension, hyperlipidemia, and albuminuria are indicated as risk factors for the development of DR [2–7]. DR based on the absence or present of neovascularization is composed of two stages: an earlier non-proliferative DR (NPDR) stage and a later proliferative DR (PDR) stage. The PDR stage is characterized by ischemia-induced and inflammation-induced neovascularization, coupled with fibrotic responses within the retina and vitreous gel. In addition, it is assumed that retinal vascular endothelial cells are involved in recruitment and proliferation of the PDR stage.

Circulating endothelial cells (CECs) detach from endothelial cells of injured endothelium and circulate in the peripheral blood [8]. The levels of CECs and the amount of apoptotic CECs have been reported to represent the degree of endothelial damage [9, 10]. Circulating endothelial progenitor cells (EPCs) are mobilized from bone marrow, and they are believed to play an important role in blood vessel repair and aid in reperfusion of the ischemic area [11]. Previous studies have indicated that circulating EPCs have altered the number of and complications in patients with type 1 and type 2 diabetes (T1D and T2D, respectively) [12–15]. Since diabetes is extremely damaging to the retinal capillary endothelium, circulating EPCs may play an important role in vascular reperfusion and retinal tissue regeneration. Nevertheless, conflicting results regarding EPC levels in diabetic patients and patients with DR have been reported. When compared with patients with no to mild DR or healthy controls, previous studies have shown that EPC levels have either reduced or increased among patients with severe DR [16–21].

Previously, we identified a genetic association for susceptibility to DR in *Rho GTPase Activating Protein 22 (ARHGAP22)* [22], which encodes negative regulators of Rho GTPase ras-related C3 botulinum toxin substrate 1 (Rac1). Additionally, it is involved in the signal transduction pathway that regulates endothelial cell capillary tube formation during angiogenesis [23]. Expression levels of ARHGAP22 play an important role in determining the mode of tumor cell movement [24]. A recent report also suggested that ARHGAP22 is associated with an increased risk of T2D and may function as an insulin regulator [25]. ARHGAP22 is an insulin-responsive and 14-3-3 binding protein; it has been reported that insulin, glucose, and growth factors, such as the platelet-derived growth factor, increase 14-3-3 protein binding to ARHGAP22, which in turn facilitates modulation of Rac1 activity [26]. Further, transcriptional activation of Rac1 has been found in the retina during the early stages of DR [27]. Previous reports have indicated that Rac1, through nicotinamide adenine dinucleotide phosphate (NADPH) oxidase activation, is pivotal in hyperglycemia-induced apoptosis in cardiomyocytes [28, 29]. A recent report suggested that the interaction between advanced glycation end products and their receptors was induced by NADPH oxidase/Rac1 activation and resulted in the activation of the C-jun N terminal kinase pathway, leading to EPCs apoptosis and dysfunction [30].

Although endothelial cell damage is a hallmark of DR, it is difficult to measure retinal local endothelial cell damage in peripheral blood circulation using CECs and apoptotic CECs as markers for endothelial injury. To examine the recruitment of EPCs from bone marrow, the most crucial marker for repair of the damaged vessel and promotion of angiogenesis, we measure the EPC levels first in this population. Therefore, we focused on assessing the relationship between EPC levels and ARHGAP22 expression in T2D patients with DR. Here, we recruited T2D patients with DR to investigate the relationship between ARHGAP22 and Rac1 expression, and EPC levels, stratified by DR severity.

## RESULTS

### Demographic and clinical characteristics of the study subjects

Fifty T2D patients with ophthalmologist-diagnosed DR were enrolled in the study; 21 (42.0%) and 29 (58.0%) had NPDR and PDR, respectively. There were no significant differences in sex, age at diagnosis, duration of diabetes, hemoglobin A1c (HbA1c) values, body mass index (BMI), systolic blood pressure (SBP), diastolic blood pressure (DBP), and smoking status between the patients with NPDR and PDR (Table 1, *P* > 0.05).

**Table 1:** Characteristics and clinical profiles of the study subjects

	T2D subjects^‡^		*p*-value^*^
	with NPDR	with PDR	
	*n* = 21	*n* = 29	
Sex			
Male	12 (57.1%)	16 (55.2%)	0.890^†^
Female	9 (42.9%)	13 (44.8%)	
Age at diagnosis T2D (mean ± SD, years)	46.3 ± 15.1	46.2 ± 9.6	0.967
Duration of diabetes (mean ± SD, years)	16.5 ± 10.0	16.3 ± 9.7	0.943
HbA_1C_ (%)	8.5 ± 1.3	8.6 ± 2.2	0.884
BMI (kg/m^2^)	24.4 ± 2.9	26.5 ± 5.2	0.108
Systolic blood pressure (mmHg)	144.5 ± 16.5	145.2 ± 16.4	0.886
Diastolic blood pressure (mmHg)	82.9 ± 9.7	81.7 ± 10.5	0.691
Smoking status			
Non smoker	16 (76.2%)	22 (75.9%)	0.979^†^
Smoker	5 (23.8%)	7 (24.1%)	

T2D, type 2 diabetes; DR, diabetic retinopathy; HbA_1C_, hemoglobin A_1C_.

^*^Student’s *t*-test; ^†^Chi-square test; ^‡^According to the American Academy of Ophthalmology proposed international scales for severity of clinical diabetic retinopathy.

### Number of EPCs in patients with DR

**Figure 1:**
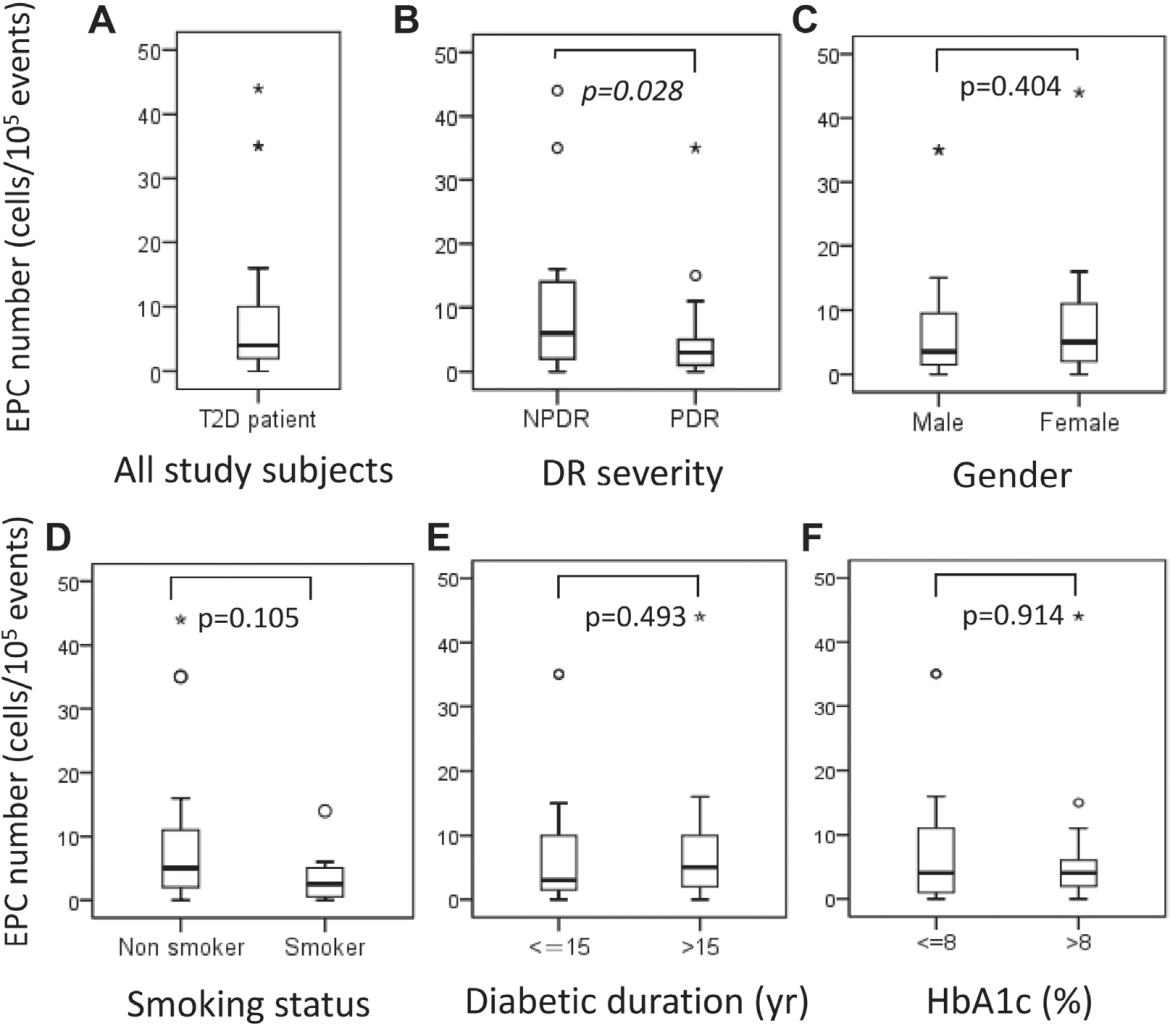
(**A**) Number of endothelial progenitor cells (EPCs) in all study subjects, and number of EPCs stratified by (**B**) diabetic retinopathy severity, (**C**) sex, (**D**) smoking status, (**E**) diabetic duration ≤ 15 years versus > 15 years, and (**F**) hemoglobin A_1C_ level ≤ 8% versus 8%. Asterisks and circles in the plot represent the extreme outlier values and outlier values, as defined by the box blot, respectively.

The median number of circulating EPCs in T2D patients was 4.0 cells/10^5^ events (interquartile range [IQR] 1.75–10.25; Figure 1A). According to DR severity, the median number of circulating EPCs was significantly lower in patients with PDR than in patients with NPDR (NPDR, 6.0 cells/10^5^ events [IQR 2.0–15.0] vs. PDR, 3.0 cells/10^5^ events [IQR 1.25–5.75]; *P* = 0.028; Figure 1B). Nevertheless, the median number of EPCs did not significantly differ when stratified by sex, smoking status, duration of diabetes (≤ 15 years vs. > 15 years), and HbA1c (≤ 8% vs. > 8%) (*P* > 0.05; Figure 1C–1F).

### The relationship between ARHGAP22 and Rac1 expression and EPC levels in patients with DR

**Figure 2:**
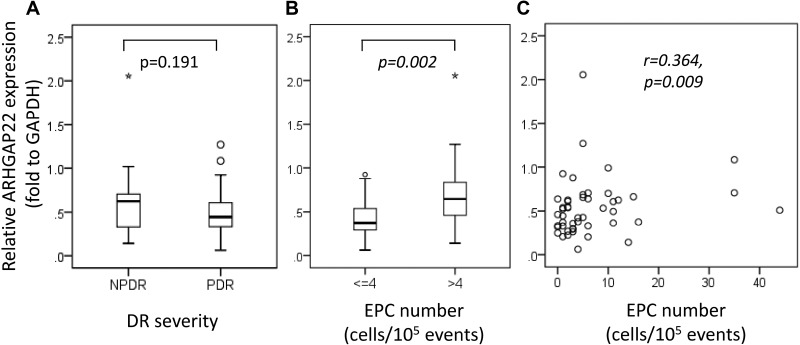
Rho GTPase activating protein 22 (ARHGAP22) expression stratified by (**A**) diabetic retinopathy severity, (**B**) number of endothelial progenitor cells (EPCs) (≤ 4 vs. > 4 cells/10^5^ events), and (**C**) the relationship between the number of EPCs and ARHGAP22 expression. Asterisks and circles in the plot represent the extreme outlier values and outlier values, as defined by the box blot, respectively.

**Figure 3:**
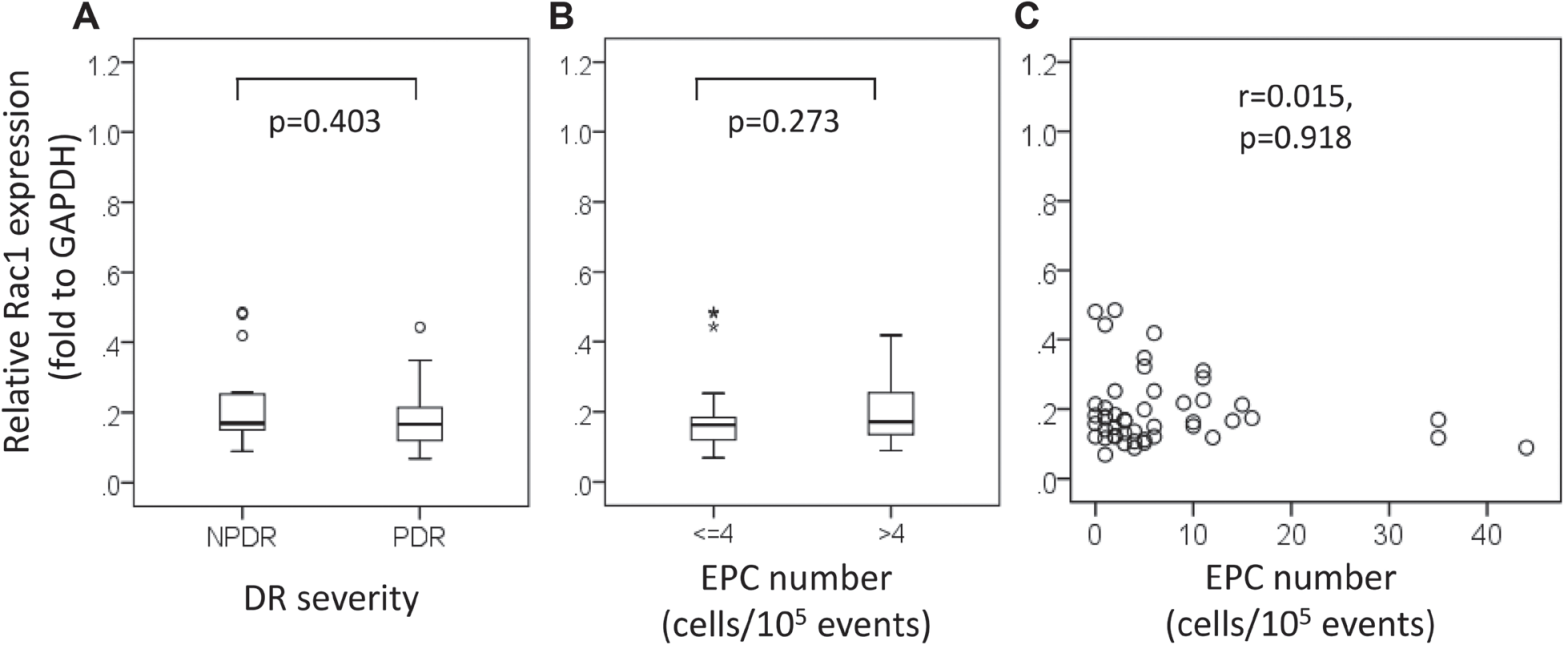
Ras-related C3 botulinum toxin substrate 1 (Rac1) expression stratified by (**A**) diabetic retinopathy severity, (**B**) number of endothelial progenitor cells (EPCs) (≤ 4 vs. > 4 cells/10^5^ events), and (**C**) the relationship between the number of EPCs and Rac1 expression. Asterisks and circles in the plot represent the extreme outlier values and outlier values, as defined by the box blot, respectively.

The relative ARHGAP22/glyceraldehyde-3-phosphate dehydrogenase (GAPDH) expression was elevated about 40% in patients with NPDR compared to patients with PDR (NPDR, 0.62 [IQR 0.32–0.71] vs. PDR, 0.44 [IQR 0.33–0.61]; *P* = 0.191; Figure 2A). We found that ARHGAP22 expression was significantly higher in patients with higher EPC levels than in patients with lower EPC levels (EPC > 4 cells/10^5^ events, 0.65 [IQR 0.44–0.90] vs. EPC ≤ 4 cells/10^5^ events, 0.37 [IQR 0.29–0.54]; *P* = 0.002; Figure 2B). In addition, the EPC levels were positively correlated with the ARHGAP22 expression levels (r = 0.364, *P* = 0.009; Figure 2C). There were no significant differences in relative Rac1/GAPDH expression between T2D patients with NPDR and those with PDR (NPDR, 0.17 [IQR 0.15–0.25] vs. PDR, 0.17 [IQR 0.12–0.22]; *P* = 0.403; Figure 3A), and patients with higher EPC levels and those with lower EPC levels (EPC ≤ 4 cells/10^5^ events, 0.16 [IQR 0.12–0.19] vs. EPC > 4 cells/10^5^ events, 0.17 [IQR 0.13–0.26]; *P* = 0.273; Figure 3B). Additionally, there was no linear relationship between EPC levels and Rac1 expression (r = 0.015, *P* = 0.918; Figure 3C).

## DISCUSSION

In the present study, we found that T2D patients with severe DR, namely PDR, had significantly lower EPC levels than those with NPDR. Additionally, EPC levels were positively correlated with ARHGAP22 expression.

Circulating EPCs are involved in angiogenesis of ischemic tissues, including retinal ischemia during DR development [31]. High levels of circulating EPCs seemed to be associated with less risk of DR progression [21]. Based on previous reports, there was reduction and dysfunction of circulating EPCs in patients with diabetes, particularly in patients with vascular complications [18, 19, 32]. However, both high and low levels of circulating EPCs have been reported in patients with NPDR and PDR compared with patients without DR or with healthy controls [16, 17, 19, 20]. Therefore, the precise role of EPCs in DR remains to be determined. In the present study, we observed that EPC levels were lower in patients with PDR than in patients with NPDR. EPC deficiencies could, theoretically, impair tissue regeneration by weakening the repair of retinal vessels during the PDR stage. In addition, several factors seem to influence the level of EPCs, including age, sex, physical activity, hypertension, smoking habits as well as the medication for diabetes. A previous study also indicated that poor glycemic control (elevated HbA1c level) may be associated with a reduction in the numbers of circulating EPCs [13]. In our study, the distributions of sex, age, HbA1c level, SBP, DBP, and smoking status were similar between the NPDR and PDR groups. However, the medication for diabetes may vary between the NPDR and PDR groups; hence, its effect on the level of EPCs should be addressed in the future. In addition, 42.1% (8/19) of patients with PDR in our study received intravitreal injections of anti-vascular endothelial growth factor (VEGF) therapy; the median number of EPCs was lower in those who did not receive anti-VEGF therapy (number of EPCs with anti-VEGF therapy, 2.5 cells/10^5^ events [IQR 0.25–4.75] vs. number of EPCs without anti-VEGF therapy, 3.0 cells/10^5^ events [IQR 2.0–6.0]). An intravitreal injection of anti-VEGF medication may not affect the differentiation and mobilization of EPCs from bone marrow into the peripheral circulation. For this reason, the results showed a lower EPC level in the PDR stage, which should be interpreted cautiously.

*ARHGAP22* is a susceptibility gene we first reported as associated with T2D patients, particularly among DR patients with PDR [22]. ARHGAP22 is a Rho GTPase-activating protein that acts as a GTPase activator for Rac1 by converting it to an inactive GDP-bound state; it is involved in the signal transduction pathway that regulates endothelial cell capillary tube formation during angiogenesis [23]. Previous reports have indicated that when ARHGAP22 expression was suppressed, Rac1 activity (the Rac1-GTP level) increased by about 2-fold in the *vitro* study [24]. These findings suggested that lower ARHGAP22 expression may cause higher Rac1 activity in the neovascularization PDR stage. It also reported that Rac1 increases vascular permeability and inflammation; inhibition of Rac1 may resolve diabetes induced arterial endothelial dysfunction [33–35]. In the present study, we observed that increased ARHGAP22 expression was positively correlated with EPC levels, but we did not observe a difference in ARHGAP22 expression based on DR severity. The possible reasons for the trend of relative ARHGAP22/GAPDH expression without a significant difference might be due to the inadequate sample size. Alternatively, ARHGAP22 may not be playing a major role in modulating EPC levels, although ARHGAP22/GAPDH expression is positively correlated with EPC levels. In addition, we did not observe a difference in Rac1 expression based on DR severity and circulating EPC levels. Further studies are necessary to investigate the relationship between Rac1-GTP (active form) expression and EPC levels. Furthermore, ARHGAP22 may also play a role in transcription regulation via its interaction with vascular endothelial zinc finger 1, by regulating activity of the endothelin-1 promoter [36]. Endothelin-1 is indicated to be involved in the regulation of EPC recruitment. Elevated plasma levels of endothelin-1 have been reported in T2D patients, thus suggesting that they contribute to endothelial dysfunction in these patients [37–39].

Several limitations of the present study need to be acknowledged. The number of the study subjects was limited; larger cohort studies should be conducted enrolling DR patients with varying degrees of severity in order to investigate the relationship between EPC levels and ARHGAP22 expression levels. We found that the change in EPC levels correlated with ARHGAP22 expression in current study; however, we could not determine whether the function changes of the EPCs differed according to the ARHGAP22 level. Isolating individuals circulating EPCs in order to test the EPCs function such as the capacity of the colony to form or the adhesive need should be performed in future studies. Based on the current data, the number of EPCs could be a biomarker for DR severity, enabling the development of a personalized follow-up strategy [40]. The development of therapeutics targeting EPCs or EPC-related signaling for DR is promising.

This is the first study to report the relationship between ARHGAP22 expression and circulating EPC levels. Higher expression levels of ARHGAP22 were associated with higher levels of EPC in this cohort of T2D patients with DR. ARHGAP22 may possibly be involved in mobilizing or activating EPCs, thus contributing to neovascularization during DR development.

## MATERIALS AND METHODS

### Study subjects

We recruited unrelated T2D subjects with DR, who were recruited from the China Medical University Hospital (CMUH) in Taichung, Taiwan from November 2014 to January 2015. All subjects underwent a complete ophthalmologic examination, including corrected visual acuity, funduscopic examination, and fundus photography. The examinations were graded by an expert ophthalmologist according to the international scales for severity of clinical DR proposed by the American Academy of Ophthalmology [41]. For each patient, information regarding sex, current age, age at diabetes diagnosis, and smoking history were collected. SBP, DBP, BMI, and HbA1c levels were determined. Patients with retinal pathologies other than DR, including those who received panretinal photocoagulation laser treatment and vitrectomy, and those with clinically significant macular edema in the past 6 months were excluded. In addition, patients were excluded if they had a history of chronic heart disease, impaired hepatic function, and any type of cancer. This study was approved by CMUH’s institutional review board, and informed consent was obtained from all study participants.

### Endothelial progenitor cells analysis

**Figure 4:**
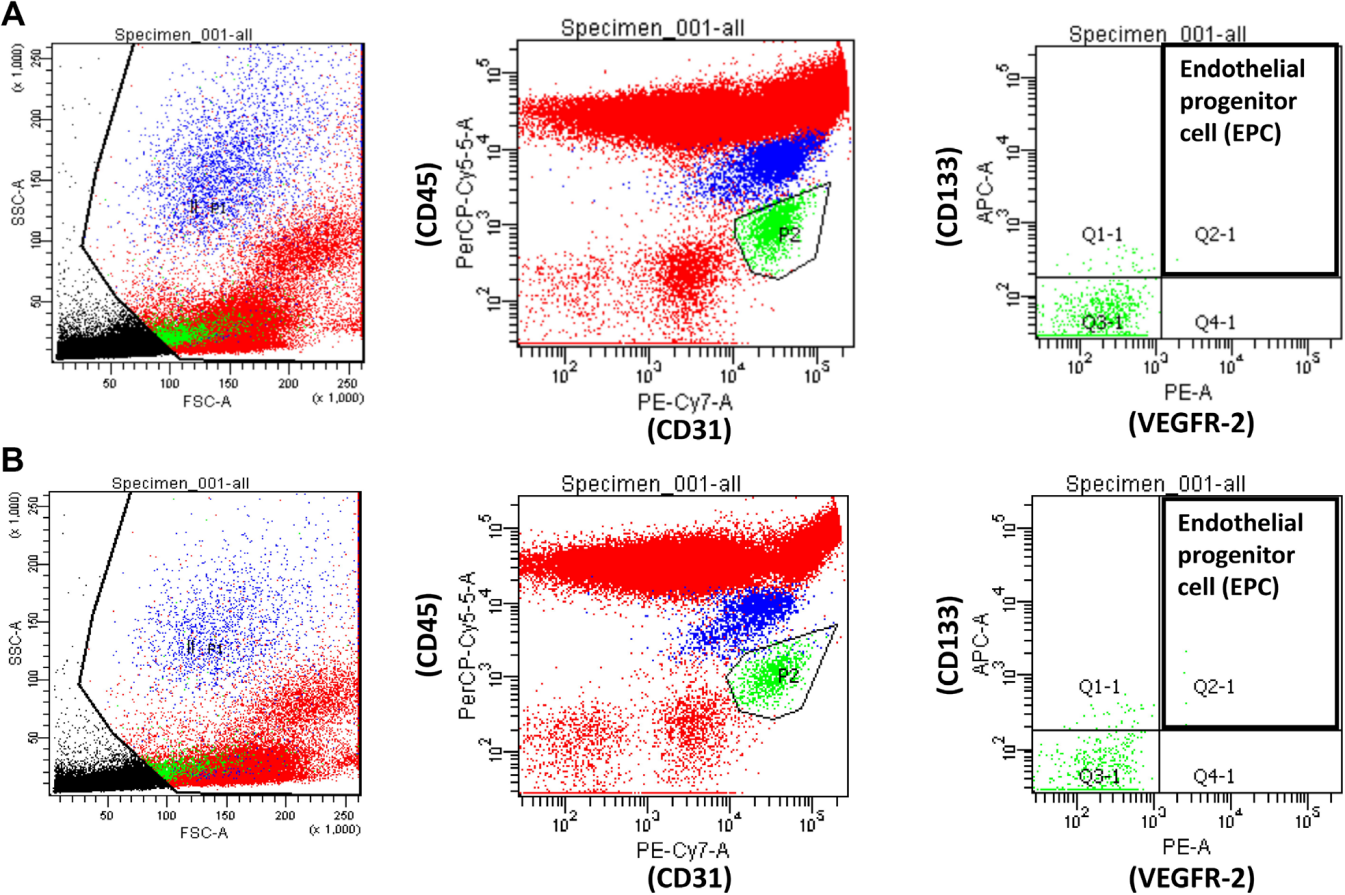
Representative data from a patient with (**A**) non-proliferative diabetic retinopathy (DR) and (**B**) proliferative DR for identification and quantification of circulating endothelial progenitor cells by flow cytometric analysis of staining for CD31^+^/vascular endothelial growth factor-2^+^/CD45^dim^/CD133^+^.

Circulating EPCs were quantified using the method devised by Duda *et al.* [42], with a few modifications described in our previous study [43]. Flow cytometry was used for whole blood analysis without enrichment procedures to avoid manipulation artifacts. The EPCs were characterized as CD31^+^/vascular endothelial growth factor-2 (VEGFR-2)^+^/CD45^dim^/CD133^+^. The chromophores conjugated with specific antibodies used in this study included CD31-FITC (BD Pharmingen, San Diego, CA), VEGFR2-PE (BD Pharmingen), CD45-PerCP (BD Pharmingen), and CD133-PE (Miltenyi Biotec, Auburn, CA). During the analysis of flow cytometry data, the mononuclear cell population was gated to avoid red blood cell, platelet, cell debris, and neutrophil contamination; 100,000 events in the gated population were collected using a FACSCanto flow cytometer (BD Biosciences, San Jose, CA, USA). The acquisition data were collected and analyzed using CellQuest Software (BD Biosciences). Representative data for identification and quantification of EPCs by flow cytometric analysis is shown in Figure 4.

### RNA preparation and quantitative real-time polymerase chain reaction

Total RNA from whole blood was extracted using the NucleoSpin RNA Blood kit (Macherey-Nagel, Düren, Germany) according to the manufacturer’s instructions, and stored at –80°C. RNA quality and quantities were measured using a NanoDrop ND-1000 spectrophotometer (Thermo Fisher Scientific, Waltham, MA, USA). The 0.8 μg of total RNA was reverse transcribed using the High-Capacity cDNA Reverse Transcription kit (Applied Biosystems, Foster City, CA) according to the manufacturer’s instructions. The quantitative reverse transcriptase-polymerase chain reaction (qRT-PCR) was performed using the LightCycler 480 Real-Time PCR System (Roche, Mannheim, Germany) in 96 well plates following the manufacturer’s instruction. Gene expression of ARHGAP22 was analyzed as the relative abundance level after normalizing with the expression level of GAPDH. Primer pairs and probes were designed using the online ProbeFinder Assay Design Software (Roche). The primers for qRT-PCR were: ARHGAP22, forward; 5′-ATGCTGAGCCCAAAGATCAG-3′, reverse; 5′-CTCCCCCATCACTAGGCTTT-3′, Rac1 forward; 5′-CTGATCAGTTACACAACCAATGC-3′, reverse; 5′′- CATTGGCAGAATAATTGTCAAAGA-3′ and GAPDH, forward; 5′-AGCCACATCGCTGAGACA-3′, reverse; 5′-GCCCAATACGACCAAATCC-3′. The Universal Probe Library probes #79, #80, and #60 were designed for ARHGAP22, Rac1, and GAPDH, respectively (Roche Applied Science, Penzberg, Germany). Each reaction was performed in triplicate and repeated three times independently.

### Statistical analysis

Statistical analysis was performed using IBM SPSS Statistics 22 (IBM Co., USA). Continuous data are presented as medians and IQR, and categorical data are presented as frequencies and proportions. We conducted *t*-tests and Mann-Whitney *U* tests for continuous variables as well as chi-square tests or Fisher exact tests for categorical variables. We than divided patients with DR into high and low level EPC groups based on the median EPC value in order to analyze the relationship between circulating EPC levels and the expression of ARHGAP22; Spearman correlation tests were used to determine the correlation between EPC levels and ARHGAP22 and Rac1 expression. *P*-values less than 0.05 were considered statistically significant.

## Abbreviations

ARHGAP22: Rho GTPase Activating Protein 22
BMI: body mass index
DR: Diabetic retinopathy
EPC: endothelial progenitor cells
GAPDH: glyceraldehyde-3-phosphate dehydrogenase
HbA_1C_: hemoglobin A_1C_
NADPH: nicotinamide adenine dinucleotide phosphate
NPDR: non-proliferative DR
PDR: proliferative DR
Rac1: ras-related C3 botulinum toxin substrate 1
SBP: systolic blood pressure
T2D: type 2 diabetes
VEGF: vascular endothelial growth factor
